# Urgent Call to Ensure Clean Air in South Asia – A Growing But Neglected Public Health Emergency

**DOI:** 10.3389/ijph.2024.1607461

**Published:** 2024-05-30

**Authors:** Shuvojit Kumar Kundu, Zaki Farhana, Anton Abdulbasah Kamil, Mohammad Meshbahur Rahman

**Affiliations:** ^1^ Directorate General of Health Services, Ministry of Health and Family Welfare, Government of the People’s Republic of Bangladesh, Dhaka, Bangladesh; ^2^ Credit Information Bureau, Bangladesh Bank, Dhaka, Bangladesh; ^3^ Department of Business Administration, Istanbul Gelisim University, Istanbul, Türkiye; ^4^ Department of Biostatistics, National Institute of Preventive and Social Medicine (NIPSOM), Dhaka, Bangladesh

**Keywords:** air pollution, health, public health emergency, call to action, South Asia

## Introduction

Across its eight countries, South Asia is home to one-fourth of the world’s population. The expansion of this highly populated area is being pursued at the expense of the health and welfare of its residents, particularly the most vulnerable, due to environmental degradation. Globally air pollution is thought to be the primary cause of increased morbidity and death from cardiorespiratory disorders [1]. In south Asia, the scenario is devastating, where 29 out of 30 most polluted cities are from Bangladesh, India, and Pakistan [2]. As a results, the World Meteorological Organization has issued a “red alert” for Bangladesh, India, and Pakistan about global warming indicators [3]. The risk to the lives and health of over a billion people is demonstrated by the extended exposure to harmful air quality in various parts of this regions [3]. Due to several factors, such as home and place of employment, larger populations, high exposures, and increasing numbers of people affected by chronic diseases, people in lower socioeconomic classes in South Asia are more vulnerable to the negative effects of air pollution exposure [4]. In every nation in South Asia, air pollution is a major problem. However, the fact that things are deteriorating and weakening from the inside is frequently overlooked.

**FIGURE 1:**
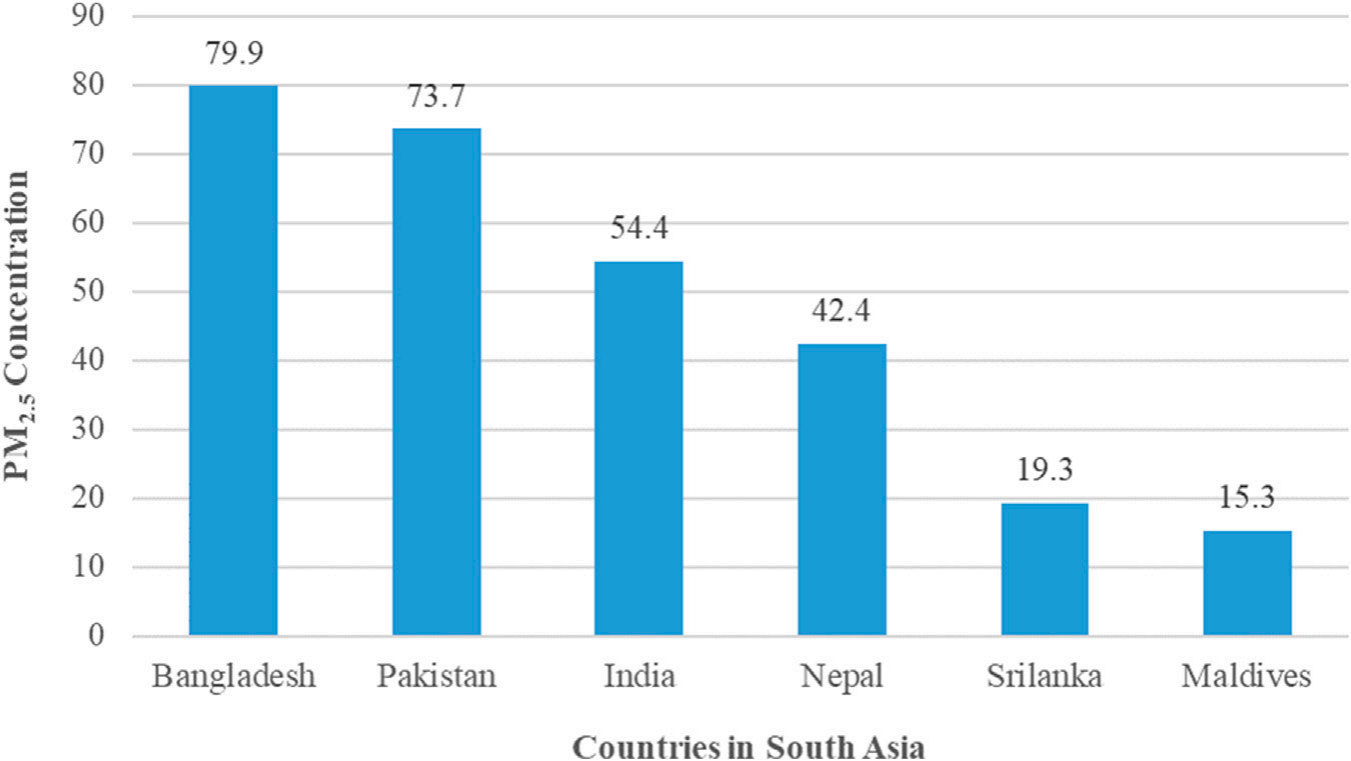
Population weighted annual average PM_2.5_ concentration in the countries of South Asia in 2023. The data reported in Figure 1 were extracted from the website of IQAir [2].

Despite being a worldwide issue, air pollution disproportionately affects people in developing countries, especially the most vulnerable groups like women, children, and the elderly [5]. In South Asia, air pollution is the second most significant risk factor for negative health consequences [5]. Rapid urbanization and industrialization are key factors behind the substantially higher air pollution levels, including particulate matter (PM) concentration, in developing countries compared to developed ones [6]. In 2019, less than 1% of the global population resided in areas that complied with the air quality guidelines 2021 of the World Health Organization (WHO) [7]. According to the guideline, the annual mean PM_2.5_ concentration for clean air quality should be at or below 5 μg/m^3^ and NO_2_ level should be at or below 10 μg/m^3^ [8]. But in South Asian countries, the values are extremely higher than the normal level as presented in Figure 1. As a most polluted country in the region, Bangladesh had almost 80 μg/m^3^ PM_2.5_ annual mean concentrations which are 16 times higher than the standard average suggested by the WHO Air Quality Guidelines (AQG) and conversely Maldives a sea girt country accounts for more than 15 μg/m^3^ PM_2.5_ annual mean concentration (Figure 1).

Recent studies and extensive research programs repeatedly demonstrate that the negative impacts of air pollution are not restricted to high levels of exposure. Detrimental health consequences can occur even at extremely low concentrations of pollutants [9]. The increased PM_2.5_ concentrations in South Asian air is supposed to cause millions of new cases of asthma, chronic obstructive pulmonary disease (COPD), acute respiratory infections, lung cancer, stroke, myocardial infarction, hypertension, diabetes, dementia, and mental health disorders [10]. Exposure to fine particle outdoor air pollution is the most significant environmental risk factor for premature death worldwide [8]. In Europe in 2021, PM_2.5_ was responsible for 432,000 premature deaths, of which 253,000 occurred at levels over the recommended WHO AQG of 5 μg/m^3^ [1]. By contrast, 91% of premature deaths due to air pollution-induced environmental effects occur in low- and middle-income countries in South-East Asia [5]. Many children under the age of five in underdeveloped countries are exposed to elevated levels of PM_2.5_, which impede their cognitive development, harm lung development, increase mortality from respiratory infections, and negatively impact their mental health [6, 11, 12]. Bangladeshis would enjoy a 5.4-year longer life expectancy if World Health Organization (WHO) guidelines were followed [10].

## Delays Will Through to the Point of No Return

A recent report highlights that 42 of the 50 cities with the worst air quality are in South Asia [10]. It predicts that by 2050, altered weather patterns will impact over 800 million people and strain the economy. South Asia’s topography, economy, and population patterns make it particularly vulnerable to air pollution challenges. The economy traditionally relies heavily on agriculture, and thermal energy is the primary energy source in the region [10]. Given that air pollution is a regional issue, a regional strategy is necessary. By adopting the strategy, nations can allocate their funds more effectively, collaborate on combating climate change, and share this collective knowledge with the public, private, non-governmental, and government sectors [10]. Cross-border collaboration is urgently needed to address this challenge. The governments in South Asia must allocate budgets and adopt eco-friendly development policies to mitigate this potential public health emergency. Additionally, wealthy nations are expected to provide promised financial assistance to low-income nations to help implement essential adaptation and mitigation measures.

### Conclusion

Air pollution in South Asia engenders a public health emergency that remains inadequately addressed. Addressing this crisis necessitates heightened attention to enhance awareness and advocate for efficacious interventions. Achieving sustainable mitigation of air pollution mandates regional cooperation. Policymakers across various echelons in these nations, spanning from local to national and regional levels, must formulate tailored policies that consider pivotal factors including economic status, local meteorological conditions, industrial activities, societal behaviors, and national literacy rates.
